# Deciphering the Pharmacological Mechanisms of Taohe-Chengqi Decoction Extract Against Renal Fibrosis Through Integrating Network Pharmacology and Experimental Validation *In Vitro* and *In Vivo*

**DOI:** 10.3389/fphar.2020.00425

**Published:** 2020-04-16

**Authors:** Shanshan Zhou, Zhongzhu Ai, Weinan Li, Pengtao You, Chaoyan Wu, Liang Li, Yuanyang Hu, Yuanming Ba

**Affiliations:** ^1^ Clinical College of TCM, Hubei University of Chinese Medicine, Wuhan, China; ^2^ Faculty of Pharmacy, Hubei University of Chinese Medicine, Wuhan, China; ^3^ Nephrology Department, Hubei Provincial Hospital of TCM, Wuhan, China; ^4^ Hubei Provincial Academy of Traditional Chinese Medicine, Hubei Provincial Hospital of TCM, Wuhan, China; ^5^ Traditional Chinese Medicine Department, Zhongnan Hospital of Wuhan University, Wuhan, China

**Keywords:** n-butanol extract of Taohe-Chengqi decoction, traditional Chinese medicine, renal fibrosis, network pharmacology, mechanism

## Abstract

Taohe-Chengqi decoction (THCQ), a classical traditional Chinese medicinal (TCM) formula, has been extensively used for treating chronic kidney disease (CKD). However, the biological activity and mechanisms of action of its constituents against renal fibrosis have not yet been investigated thoroughly. This study was aimed at devising an integrated strategy for investigating the bioactivity constituents and possible pharmacological mechanisms of the n-butanol extract of THCQ (NE-THCQ) against renal fibrosis. The n-butanol extract of THCQ was prepared by the solvent extraction method. The components of NE-THCQ were analyzed using UPLC-Q/TOF-MS/MS techniques and applied for screening the active components of NE-THCQ according to their oral bioavailability and drug-likeness index. Then, we speculated the potential molecular mechanisms of NE-THCQ against renal fibrosis through pharmacological network analysis. Based on data mining techniques and topological parameters, gene ontology, and pathway enrichment, we established compound-target (C-T), protein-protein interaction (PPI) and compound-target-pathway (C-T-P) networks by Cytoscape to identify the hub targets and pathways. Finally, the potential molecular mechanisms of NE-THCQ against renal fibrosis, as predicted by the network pharmacology analyses, were validated experimentally in renal tubular epithelial cells (HK-2) *in vitro* and against unilateral ureteral obstruction models in the rat *in vivo*. We identified 26 components in NE-THCQ and screened seven bioactive ingredients. A total of 118 consensus potential targets associated with renal fibrosis were identified by the network pharmacology approach. The experimental validation results demonstrated that NE-THCQ might inhibit the inflammatory processes, reduce ECM deposition and reverse EMT *via* PI3K/AKT/mTOR and HIF-1α/VEGF signaling pathways to exert its effect against renal fibrosis. This study identified the potential ingredients of the NE-THCQ by UPLC-Q/TOF-MS/MS and explained the possible mechanisms of NE-THCQ against renal fibrosis by integrating network pharmacology and experimental validation.

**Graphical Abstract:**
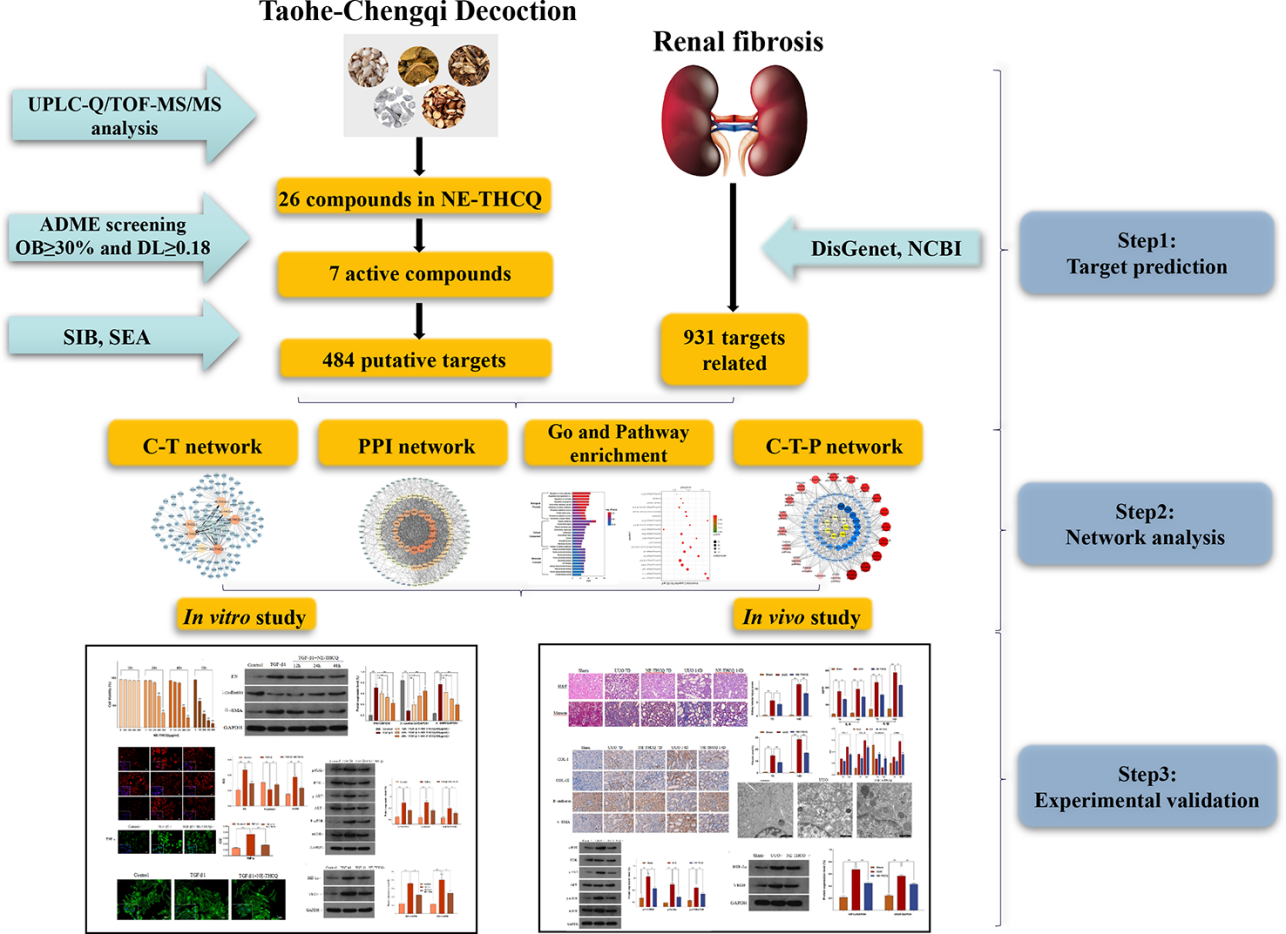
The graphical abstract of this study.

## Introduction

Renal fibrosis is the final pathway common to various chronic and progressive nephropathies to end-stage renal disease (ESRD). Renal fibrosis indicates the morphological manifestation of a continuous coordinated response of glomerulus, renal tubules, blood vessels, and renal interstitium during chronic injury (Benjamin, 2018). Actually, cortical interstitial dilation reflects the renal prognosis and dysfunction in chronic kidney disease (CKD) (Risdon et al., 1968; Karl, 1992). During chronic injury in CKD, the continuous and uninhibited deposition of the fibrous matrix decelerates the tissue repair and local blood supply, finally destroys the tissue structures and functions and eventually leads to kidney failure. Chronic kidney diseases affect 10% of the global population, and 11% of patients with stage 3 CKD ultimately progress to ESRD and require dialysis or kidney transplantation (Thomas et al., 2010). CKD is the biggest risk factor of cardiovascular diseases (Marcos et al., 2017; Vasiliki et al., 2018; Júlia et al., 2019). Presently, the most common therapies for CKD recommend angiotensin-converting enzyme inhibition (ACEI) and angiotensin receptor blockers (ARB) for controlling blood pressure and sodium bicarbonate for rectifying metabolic acidosis. Despite these treatments, the prognosis of CKD is still poor, which warrants targeted drugs for treating CKD. Therefore, scientists now focus on exploring the mechanisms of renal fibrosis and developing new drugs and effective treatments to reduce the incidence of ESRD and mortality.

Traditional Chinese medicine (TCM) can provide an alternative approach for treating renal fibrosis. Many Chinese herbal medicines (e.g. *Salvia miltiorrhiza* Bunge*, Astragalus mongholicus* Bunge, and *Rheum palmatum* L.) and active components (e.g. tanshinone, salvianolic acid, and emodin) reportedly possess antirenal fibrosis effect (Wang et al., 2015; Ma et al., 2017; Zhang et al., 2018). Unlike the one drug-one target concept of Western medicine, TCM emphasizes that the human body is an organic whole. In TCM formula, multiple herbal ingredients and bioactive components target multiple receptors and produce synergistic or antagonistic effects (Zhou et al., 2019). The conventional pharmacological methods are incapable to elucidate the underlying therapeutic mechanisms of TCM.

Taohe-Chengqi decoction (THCQ), a famous formula recorded in “Treatise on Febrile Diseases” by Zhongjing Zhang in the Han Dynasty, has been listed in the first “Catalogue of ancient classical TCM formulas” issued by the State Administration of TCM of the People’s Republic of China. THCQ consists of five Chinese medicines, including *Prunus persica* (L.) Batsch *or P. davidiana* Franch., *Rheum palmatum* L., *Cinnamomum cassia* (L.) J. Presl, *Glycyrrhiza glabra* L., and Sodium sulfate. Clinically, THCQ is prescribed for different chronic kidney diseases, including chronic renal failure, chronic pyelonephritis, and diabetic nephropathy. Numerous clinical studies have demonstrated that THCQ can effectively treat chronic kidney diseases due to its antiinflammatory, lipid regulating and renal function improving properties (Zhao et al., 2012; Zhang et al., 2016; Zhang et al., 2019). However, the bioactive components of THCQ and their pharmacological mechanisms remain relatively unclear.

With the rapid development of bioinformatics, network pharmacology has emerged as a powerful tool to explore TCM (Cao et al., 2018; Ma et al., 2018; Yu et al., 2018). Based on system-biology, multi-directional pharmacology, and high-throughput analysis, network pharmacology can thoroughly explain the complicated relationship between drugs and diseases by constructing biological network and network visualization analysis of the potential active ingredients, hub targets, signaling pathways and diseases (Yuan et al., 2017; Ning et al., 2018; Huang et al., 2019). Therefore, network pharmacology can effectively explore the multi-components, multi-targets, and multi-pathways of TCM.

In the current study, a comprehensive method was used to illustrate the molecular mechanisms of THCQ. Briefly, we used UPLC-Q/TOF-MS/MS to analyze the bioactive components of the n-butanol extract from THCQ (NE-THCQ), and then network pharmacology was applied to further investigate the correlations among the active ingredients of NE-THCQ, the potential protein targets and hub signaling pathways associated with renal fibrosis. Moreover, the molecular mechanisms of NE-THCQ predicted by network pharmacology approach against renal fibrosis were validated by *in vitro* and *in vivo* experiments. The graphical abstract of this study is shown in **Graphical Abstract**.

## Materials and Methods

### Reagents and Materials

All medicinal plants were purchased from Hubei Tianji Traditional Chinese Medicine Pieces Co., Ltd (Wuhan, China). Fetal bovine serum (FBS) was purchased from BI (USA). Phosphate buffer saline (PBS) and Dulbecco’s modified Eagle medium (DMEM) was procured from Gibco (USA); penicillin-streptomycin mixture and Cell Counting Kit-8 (CCK-8) from Beijing Suolaibao Technology Co. LTD. (Beijing, China); dimethyl sulfoxide (DMSO) from Sigma (Missouri, USA); trypsin−EDTA from Thermo Fisher Scientiﬁc (USA); recombinant human TGF-β1 from Peprotech (USA).The ELISA assay kits for interleukin (IL)-6, IL-1β were all obtained from Cusabio (Beijing, China); phosphorylated (p)- AKT, AKT, and HIF-1α from Affinity Biosciences (OH, USA); COL-I, α-SMA from Boster (CA, USA); COL-III, E-cadherin, FN, p-PI3K from Abcam (MA, USA); VEGF, PI3K, mTOR, p-mTOR from Proteintech Group, Inc (Wuhan China).

### Preparation of Extracts and Subfractions

For preparing THCQ extracts, four of Chinese herbal medicines (*Prunus persica* (L.) Batsch, *Rheum palmatum* L., *Cinnamomum cassia* (L.) J. Presl, *Glycyrrhiza glabra* L.) were homogenized and extracted with eightfold of 70% ethanol for 2 h. The ethanol extracts were concentrated to dryness under vacuum, diluted with water and then partitioned by petroleum ether, chloroform, ethyl acetate, and n-butanol successively. Sodium sulfate was directly treated as an extracted part. The entire solvent was evaporated under reduced pressure to obtain petroleum ether extract, chloroform extract, ethyl acetate extract, n-butanol extract.

### UPLC-Q/TOF-MS/MS Method for Component Analysis

UPLC-Q/TOF-MS/MS analysis was performed using a Waters Xevo G2 Q/TOF (Waters, USA), including an autosampler, a diode-array detector (DAD), a micro TOF-Q mass spectrometer (Waters, USA) and an Apollo electrospray ionization (ESI) source. The analysis was conducted using an analytical Welch Ultimate TM UPLC XB-C18 column (100 mm × 2.1 mm, 1.8 µm). The mobile phase A consisted of 0.1% formic acid in H_2_O, and mobile phase B consisted of acetonitrile; the solvent gradient varied from 5% B in the first 25 min to 95% B up to 40 min at a flow rate of 0.3 ml/min; the column oven temperature was 40°C. The detection was performed at 210 nm. The acquisition of high-resolution mass spectra was conducted in the positive and negative ion modes at the dry gas flow rate of 800 L/h and the gas temperature of 200°C. The capillary voltage was 30 kV; the analysis was carried out using a scan from m/z 50 to 1,500.

### Analysis of N-Butanol Extract of THCQ (NE-THCQ) by Network Pharmacology

#### Prediction of Bioactive Ingredients in NE-THCQ

The ingredients identified by UPLC-Q/TOF-MS/MS analysis of NE-THCQ were considered as candidate compounds. The pharmacological information of the compounds was retrieved from the TCM systems pharmacology (TCMSP) database (http://tcmspw.com/) (Ru et al., 2014). Oral bioavailability (OB) and drug-like quality (DL) were selected as parameters for the preliminary screening of the collected compounds. OB denotes the percentage of an orally administered dose of unchanged drug that reaches the systemic circulation. It represents the convergence of the processes of absorption, distribution, metabolism, and excretion (ADME) (Xu et al., 2012). A high OB value is often a key indicator in determining the DL of bioactive molecules as therapeutic agents. DL is a qualitative concept used in drug design to estimate the “drug-like” qualities of a prospective compound. The DL index can be useful in optimizing pharmacokinetic and pharmaceutical properties, such as solubility and chemical stability (Christopher et al., 1997). Herein, we assigned an OB value of ≥ 30% or a DL value of ≥ 0.18, as criteria to identify the potential active ingredients (Cao et al., 2018).

#### Potential Targets Intersection of NE-THCQ With Disease

The protein targets of the active substances in NE-THCQ were searched from two databases, namely, the Swiss target prediction (SIB) and the similarity ensemble approach (SEA). The renal fibrosis-related genes were collected from the DisGeNet database and the NCBI database. The keywords, “renal fibrosis” and “chronic kidney disease,” were used, and the targets were human genes enrolled in this study. Then, the interaction of those targets collected from above was considered as potential therapeutic targets. To obtain the interaction relationship between target proteins, the STRING database was used.

#### Pathway and Functional Enrichment Analysis

The representative pathways associated with NE-THCQ against renal fibrosis was analyzed by ClueGO (Version 2.5.4) (Mikail and Olaf, 2010). The gene ontology (GO) function enrichment analysis was conducted using the database for annotation, visualization, and integrated discovery (DAVID, version 6.8) (Da et al., 2008). The analysis was based on the screened potential therapeutic targets.

#### Network Construction and Analysis

Compound-target (C-T), protein-protein interaction (PPI), compound-target-pathway (C-T-P) networks were constructed using the Cytoscape 3.6.0 software, a popular bioinformatics software package for biological network visualization and data integration (Smoot et al., 2010). In the graphical network, nodes indicate targets, compounds and pathways, and edges indicating C-T, PPI, and C-T-P interactions.

### Experimental Verification

#### Cell Line and Culture

The human proximal renal tubular epithelial (HK-2) cell line was purchased from the Wuhan Academy of Life Sciences (Wuhan, China). The cells were incubated in DMEM supplemented with 10% FBS,100 U/ml penicillin and 100 µl/ml streptomycin. The cells at approximately 60%–70% conﬂuency were digested with 0.05% trypsin−EDTA and seeded in 96-well, 12-well, or 6-well plates (Corning, NY, USA). The cells were then cultured in a serum-free medium for 12 h and stimulated with TGF-β1 (10 ng/ml) in the absence and presence of NE-THCQ.

#### CCK-8-Based Cytotoxicity Assay

The cytotoxicity of NE-THCQ was determined by CCK-8 assay. Briefly, the cells were seeded onto 96-well plates and cultured until they adhered completely. Then, cells were treated with NE-THCQ at different concentrations (0 µg/ml, 100 µg/ml, 200 µg/ml, 400 µg/ml, 600 µg/ml) for 12 h, 24 h, 48 h and 72 h, and then, 10 µl of CCK-8 was added and incubated for another 4 h. The absorbance was recorded at 450 nm, and experiments were performed parallelly in triplicate.

#### Cytoskeleton Assay

To examine cytoskeleton assembling, cultured HK-2 cells on 10 mm coverslips were fixed in a 4% formaldehyde solution for 10 min at room temperature and then permeated in 0.1% Triton X-100 in PBS for 5 min. After washing with PBS, the cells were incubated in the staining solution, containing Alexa Fluor-488 phallotoxins at 1:50 dilution for 20 min at room temperature. The samples were mounted in the antifading mounting medium, and F-actin distribution was captured under an inverted laser confocal microscope (Zeiss, Germany).

#### Establishment and Grouping of the Unilateral Ureteral Obstruction Rat Model

Male Wistar rats (n = 36), weighing 180–220 g, were purchased from the Hubei Provincial Center for Disease Control and Prevention (Wuhan, China). All animals were kept under appropriate conditions at 24°C ± 1°C temperature, 60%–70% relative humidity, and 12-h light/dark cycle. After an environmental adaptation period of 7 days, the rats were randomly divided into three groups, namely, the sham-operation group (Sham), UUO group (UUO), and UUO + NE-THCQ treatment group (NE-THCQ, 80 mg/kg, p.o.), 18 rats in each group. The UUO rats were anesthetized with chloral hydrate (30 mg/kg), and then the left ureter was exposed and ligated with 4-0 silk sutures (Ucero et al., 2013). The animals of the sham operation group were treated identically to the UUO group, except the ureters were not ligated. The rats were killed on the 7th and 14th day, respectively after UUO induction to prepare serum samples and kidney tissue specimens for further experiment. All animal experiments were conducted in accordance with the Animal Care and Use Committee of the Institute of Materia Medica, P.R. China.

#### ELISA Assay

The rats were intraperitoneally anesthetized by chloral hydrate (30 mg/kg), and the blood samples were collected from the abdominal aorta on the 7th and 14th day after UUO operation. The blood samples were kept at room temperature for 12 h, and then 3500 r centrifuged for 15 min (4°C). The supernatants were collected and stored at −80°C until used. According to the manufacturer’s protocols, the levels of IL-6 and IL-1β were detected by commercial ELISA kits.

#### Renal Pathological Examination

The obstructed kidney was fixed with paraformaldehyde (4%) (Sigma-Aldrich) and then, dehydrated in ethanol, embedded in paraffin. The 4-µm paraffin sections were prepared for the hematoxylin-eosin (H&E) and Masson’s trichrome staining. The extent of the renal injury was graded on a scale from 0 to 3 by examining tubular atrophy and necrosis, lymphocyte infiltration and interstitial fibrosis. Each criterion was scored as follows: 0 = none; 1 = mild (< 25%); 2 = moderate (25% to 50%); 3 = severe (> 50%) (Zhao et al., 2016). The degree of fibrosis was assessed by optical microscopy (Olympus) according to the quantity of collagen deposited in the renal interstitium (stained blue within the whole cortical area). The collagen was quantified using Image-Pro Plus 6.0 software (Media Cybernetics, Inc., Rockville, MD, USA). For H&E staining and Masson’s trichrome, five fields were selected from each group and observed under a magnification of 200 ×.

#### Immunohistochemistry

The paraffin-embedded kidney sections were deparaffinized in xylene and rehydrated through a descending gradient of ethanol. The sections were subsequently immersed in EDTA antigen repair buffer (pH 8.0) and then boiled for 10 min at high power in a microwave oven. After cooling naturally, the specimens were washed three times in PBS (pH 7.4), for 3 min on each occasion. The slices were incubated in 3% H_2_O_2_ at room temperature under dark conditions for 25 min and then washed in PBS (pH 7.4) three times, for 5 min on each occasion. The sections were then incubated in primary antibody overnight at 4°C and then washed three times in PBS (pH 7.4) for 5 min on each occasion. The primary antibodies used in the analysis were as follows: anti-E-cadherin (1:200 dilution), anti-α-SMA (1:100), anti-collagen-I (1:100), and anti-collagen-III (1:200). An appropriate secondary antibody (antimouse or antirabbit) was incubated with the slides at 37°C for 20 min and then washed four times in PBS for 3 min on each occasion. The sections were immunostained with 3,3-diaminobenzidine (DAB) and counterstained with hematoxylin then sealed using neutral gum. Optical microscopy (Olympus BX 41, Japan) was employed for image acquisition, and Image-Pro Plus 6.0 software was used for analysis. Brown staining was considered positive. The staining intensity was calculated from the optical density of each image.

#### Immunofluorescence study

The HK-2 cells, cultured on coverslips, were washed with PBS three times and fixed in paraformaldehyde (4%) for 15 min. After extensively washing three times with PBS, the cells were permeated with 0.5% TritonX-100 for 15 min. The cells were then blocked with goat serum for 30 min at room temperature and incubated with primary antibody FN (1:100), E-cadherin (1:100), α-SMA (1:100) overnight at 4°C. Then, the cells were stained with Cy3-labeled goat antirabbit IgG (H + L) (1:200) for 60 min at 37°C. After washing with PBS, the cells were counterstained with DAPI to visualize the cell nuclei and analyzed under a fluorescence microscope (Olympus, BX 53, Japan).

#### Transmission Electron Microscope (TEM)

The samples were fixed in 4% paraformaldehyde and 1% osmium tetroxide. After washing in double-distilled water for three times, the samples were stained with uranyl acetate and lead citrate at room temperature. After air-drying, the sections were examined under a transmission electron microscope (TEM) (FEI, USA).

#### Western Blot Analysis

The kidney tissue and HK2 cells were lysed by RIPA buffer (Beyotime Institute of Biotechnology, China), containing cocktail protease inhibitors for 30 min on ice. The total protein concentration was determined by BCA protein assay kit (Beyotime). An equal amount of protein (30 µg) was separated using 10% SDS-PAGE and then electrophoretically transferred onto PVDF (Millipore. Billerica, MA, USA). The membranes were blotted with 5% fat-free milk in TBST buffer for 2 h at room temperature and then incubated at 4°C overnight with primary antibodies: anti-FN (1:1,000), anti-E-cadherin (1:5,000), anti-α-SMA (1:2,000), anti-PI3K (1:1,000), anti-p-PI3K (1:800), anti-AKT (1:1,000), anti-p-AKT (1:1,000), anti-mTOR (1:1,000), anti-p-mTOR (1:1,000), anti-HIF-1α (1:1,000) and anti-VEGF (1:1,000). Then, the membranes were incubated with HRP-conjugated anti-rabbit/mouse IgG. The blots were imaged under an enhanced chemiluminescence (ECL) system. The target band molecular weights and net optical density were analyzed using AlphaEase FC software (Alpha Innotech, USA).

### Statistical Analysis

All data were expressed as the mean ± standard deviation (SD). Graphpad Prism 7 software was used to ascertain statistically significant differences. The differences among multiple groups were evaluated using the one-way analysis of variance (ANOVA). The difference between the means was considered statistically significant at *P* < 0.05.

## Results

### Identification of Bioactive Components in NE-THCQ

**Figure 1:**
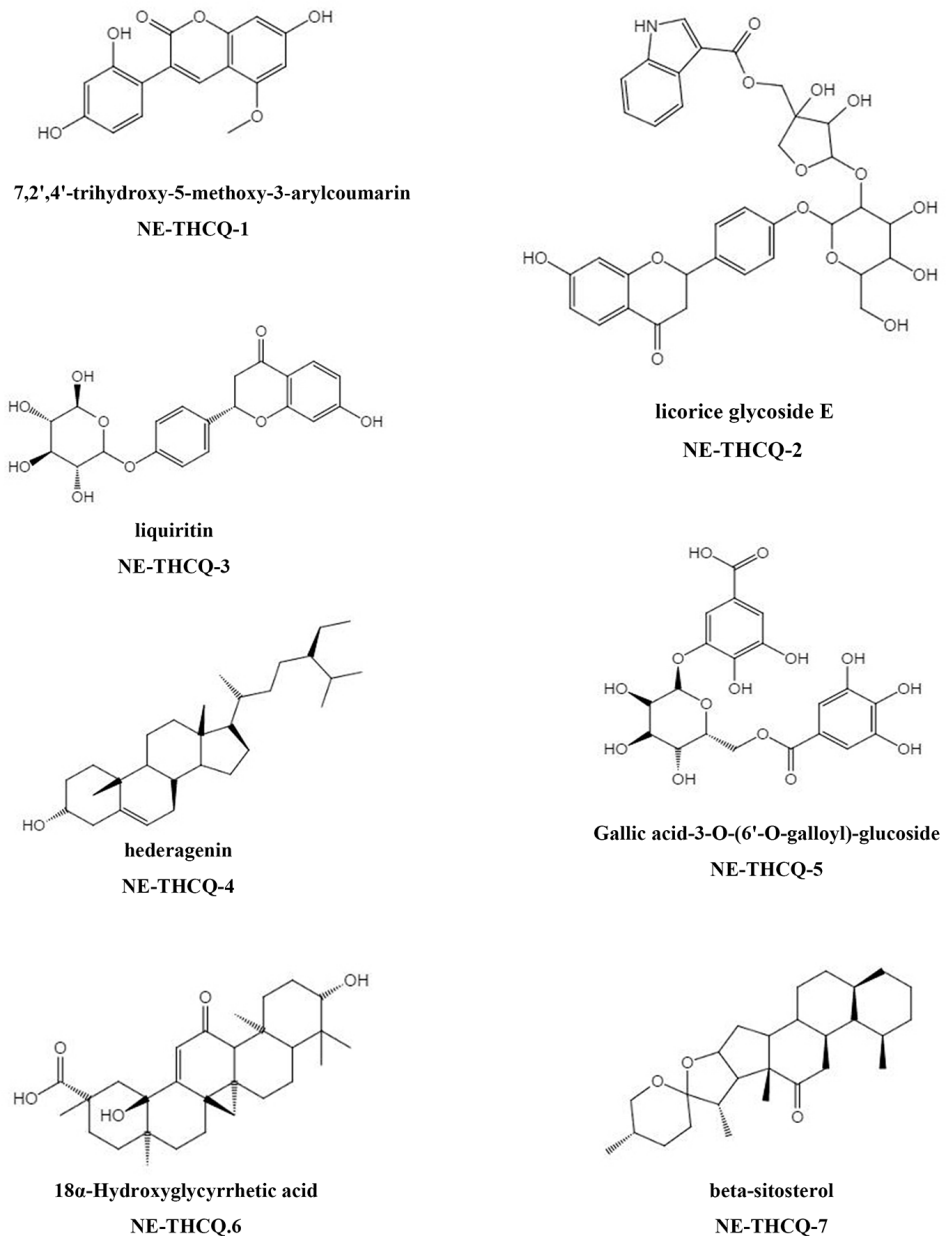
The molecular structures for bioactive components of n-butanol extract of Taohe-Chengqi decoction (NE-THCQ).

The phytochemical composition of NE-THCQ was evaluated using UPLC-Q/TOF-MS/MS, and the total ion chromatogram is showed in **Supplementary Figure 1**. In **Table 1**, approximately 26 chromatographic peaks were identified in the chemical profile of NE-THCQ. The physicochemical characteristics of the compounds analyzed by UPLC-Q/TOF-MS/MS were obtained from the TCMSP database. Among the 26 compounds in NE-THCQ (**Table 1**), 7 met the requirements of OB ≥ 30% and DL index ≥ 0.18; they were 7, 2′, 4′-trihydroxy-5-methoxy-3-arylcoumarin (NE-THCQ-1), licorice glycoside E (NE-THCQ-2), liquiritin (NE-THCQ-3), gallic acid-3-O-(6′-O-galloyl)-glucoside (NE-THCQ-4), hederagenin (NE-THCQ-5), beta-sitosterol (NE-THCQ-6), and 18α-hydroxyglycyrrhetic acid (NE-THCQ-7). Those components were selected as the potential bioactive components for further analysis; their molecular structures are shown in **Figure 1**.

**Table 1:** Information of 26 components of n-butanol extract of Taohe-Chengqi decoction (NE-THCQ) determined by UPLC-Q/TOF-MS/MS.

No.	t_R_ (min)	Compound	Molecular formula	Parent (m/z)	Polarity	Classification	OB (%)	DL
1	5.708	Amygdalin	C_20_H_27_NO_11_	456.1502	Negative	Persicae Semen	4.42	0.61
2	9.311	Liquiritin	C_21_H_22_O_9_	417.1194	Negative	Licorice	65.69	0.74
3	9.709	Rheochrysin	C_22_H_22_O_10_	445.0759	Negative	Radix Rhei Et Rhizome	18.31	0.82
4	12.569	Schaftoside	C_26_H_28_O_16_	595.1627	Negative	Licorice	7.88	0.75
5	12.933	18α-Hydroxyglycyrrhetic acid	C_30_H_46_O_4_	417.1194	Negative	Licorice	41.16	0.71
6	15.294	Catechin-pentaacetate	C_25_H_24_O_11_	499.0830	Negative	Radix Rhei Et Rhizome	27.58	0.77
7	15.672	Chrysophanol glucoside	C_21_H_20_O_9_	415.1021	Negative	Radix Rhei Et Rhizome	20.06	0.76
8	15.672	Gallic acid-3-O-(6′-O-galloyl)-glucoside	C_20_H_20_O_14_	483.0894	Negative	Radix Rhei Et Rhizome	30.25	0.67
9	20.536	2-Methyl-5-carboxymethyl-7-hydroxychromanone	C_12_H_12_O_5_	235.9244	Negative	Radix Rhei Et Rhizome	14.80	0.12
10	20.536	1,8-Dihydroxy-3-methoxy-2,6-dimethyl-9,10-anthraquinone	C_17_H_14_O_5_	297.0393	Negative	Radix Rhei Et Rhizome	5.53	0.29
11	22.034	Glycyram	C_42_H_62_O_16_	822.3981	Negative	Licorice	19.62	0.11
12	24.880	Licorice glycoside E	C_35_H_48_O_14_	692.3702	Negative	Licorice	32.89	0.27
13	5.985	1-O-Galloylpedunculagin	C_41_H_28_O_26_	937.3089	Positive	Radix Rhei Et Rhizome	38.09	0.04
14	12.623	Licuraside	C_26_H_30_O_13_	551.1746	Positive	Licorice	5.25	0.77
15	13.399	Syringaldehyde	C_9_H_10_O_4_	182.9843	Positive	Cinnamomi Ramulus	67.06	0.05
16	13.467	7,2′,4′-Trihydroxy-5-methoxy-3-arylcoumarin	C_16_H_12_O_6_	301.1410	Positive	Licorice	83.71	0.27
17	14.485	Hispaglabridin B	C_25_H_26_O_4_	391.0491	Positive	Licorice	22.94	0.88
18	15.295	3-Hydroxy-25-norfriedel-3,1(10)-dien-2-one-30-oic acid	C_28_H_54_O_4_	455.0935	Positive	Radix Rhei Et Rhizome	18.40	0.78
19	15.673	18beta-glycyrrhetinic acid	C_30_H_46_O_4_	471.0323	Positive	Licorice	22.05	0.74
20	18.243	4,2′,4′, Alpha-Tetrahydroxydihydrochalcone	C_15_H_14_O_5_	275.2786	Positive	Licorice	2.45	0.16
21	19.086	Glabrolide	C_30_H_44_O_4_	469.1114	Positive	Licorice	17.46	0.61
22	21.569	Licoricesaponin G2	C_42_H_62_O_17_	840.4093	Positive	Licorice	6.39	0.11
23	22.553	Glycyrrhizin	C_42_H_62_O_16_	824.4168	Positive	Licorice	9.06	0.11
24	24.638	Artonin E	C_25_H_24_O_7_	437.1929	Positive	Licorice	11.38	0.8
25	24.760	Beta-sitosterol	C_29_H_50_O	415.2216	Positive	Licorice	36.91	0.75
26	25.036	Hederagenin	C_30_H_48_O_4_	415.2116	Positive	Persicae Semen	36.91	0.75

### Compound-Target Network Construction

**Figure 2:**
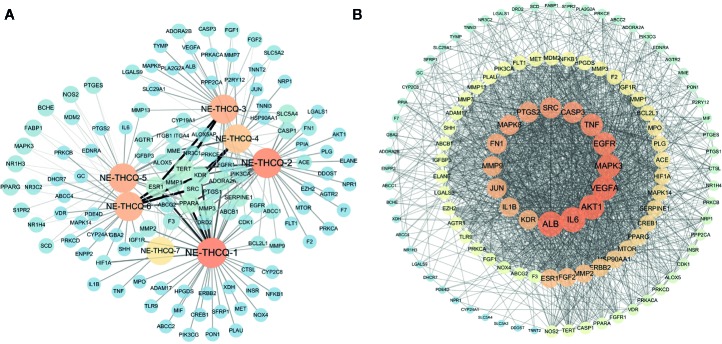
The networks of n-butanol extract of Taohe-Chengqi decoction (NE-THCQ) on antirenal fibrosis. **(A)** The compound-target network of NE-THCQ. The orange nodes represent active compounds and the blue nodes represent targets. Nodes size and color depth are proportional to their degree. Edge widths are proportional to the edge betweenness. **(B)** The protein-protein interaction (PPI) network of protein targets obtained from STRING database and constructed by Cytoscape. The colors of the nodes are illustrated from dark orange to yellow to blue in descending order of degree values.

Among the seven compounds, 484 targets were retrieved from SIB and SEA databases. A total of 931 candidate targets of renal fibrosis were obtained from NCBI and DisGeNet databases. Considering the intersection of the 931 candidate targets related to renal fibrosis and the 484 putative targets of the seven compounds in NE-THCQ, a total of 118 common targets were identified as potential therapeutic targets of NE-THCQ against renal fibrosis for constructing the compound-target (C-T) network. The C-T network was constructed using Cytoscape (**Figure 2A**), which included 125 nodes (seven for potential bioactive components and 118 for protein targets). Among these bioactive components, 7, 2′, 4′-trihydroxy-5-methoxy-3-arylcoumarin (NE-THCQ-1, degree = 43) exhibited the highest correlation with renal fibrosis targets, and the rest were licorice glycoside E (NE-THCQ-2, degree = 38), hederagenin (NE-THCQ-5, degree = 31), beta-sitosterol (NE-THCQ-6, degree = 30), liquiritin (NE-THCQ-3, degree = 30), gallic acid-3-O-(6′-O-galloyl)-glucoside (NE-THCQ-4, degree = 25), and 18α-hydroxyglycyrrhetic acid (NE-THCQ-7, degree = 19).

### PPI Network Analysis

**Figure 3:**
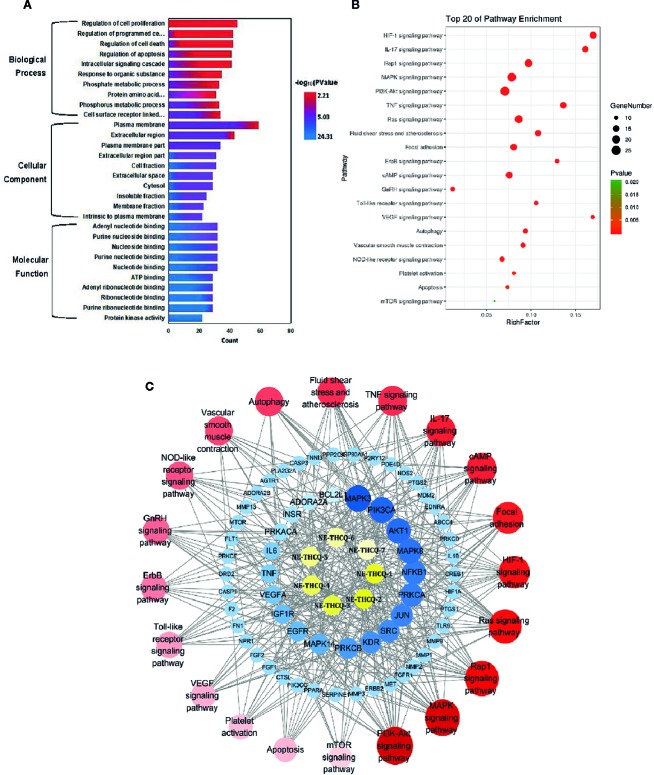
**(A)** The gene ontology (GO) enrichment analysis for key targets. **(B)** The KEGG pathway enrichment analysis of key targets. **(C)** The component-target-pathway network constructed by Cytoscape. The yellow nodes represent active components in n-butanol extract of Taohe-Chengqi decoction (NE-THCQ), the blue nodes represent putative targets, the red nodes represent the signaling pathways. Nodes size and color depth are proportional to their degree.

The 118 potential therapeutic target genes were uploaded to the STRING database, which provided the information on predicted interaction, and they were imported in Cytoscape 3.6.0 for analyzing and constructing the PPI network (**Figure 2B**). In the PPI network, targets with a higher degree played an important role in the central correlation. In **Figure 3**, top 16 genes, ranked by degree value, were collected to be the hub targets, namely, ALB, IL6, AKT1, VEGFA, MAPK3, EGFR, TNF, CASP3, SRC, PTGS2, MAPK8, FN1, MMP9, JUN, KDR, and IL1B.

### GO Enrichment Analysis

GO enrichment analysis of the 118 potential therapeutic targets was performed for identifying the relevant biological functions of NE-THCQ against renal fibrosis. The top 10 significantly enriched terms with a greater number of involved targets in biological process (BP), cellular component (CC), and molecular function (MF) categories are shown in **Figure 3A**, which indicates that NE-THCQ may regulate cell proliferation, apoptosis, and migration *via* transition metal ion binding, nucleoside and nucleotide binding to the plasma membrane, extracellular region, and cytosol to exert its therapeutic effects against renal fibrosis.

### Pathway Enrichment and Compound-Target-Pathway Network Construction

To explore the potential pathways of NE-THCQ on renal fibrosis, the pathway enrichment of the 118 potential therapeutic targets was performed. The top 20 significantly enriched pathways are shown in **Figure 3B**. Among these potential pathways, PI3K/AKT signaling was the most prominently enriched pathway according to the gene numbers; HIF-1α, TNF, VEGF, and mTOR signaling pathways were also included, which were categorized for inflammation, hypoxia and angiogenesis, proliferation, and migration. As genes cannot exhibit their biological and pharmacological activities independently, a compound-target-pathway network was established based on the top 20 signaling pathways and the involved targets and compounds to further elucidate the molecular mechanism of NE-THCQ against renal fibrosis (**Figure 3C**). After integrating drug target prediction, pathway and function enrichment, and network analyses, we identified AKT1, VEGFA, TNF, IL6, HIF-1α, IL-1β, mTOR, and FN1 as relatively high-relevant targets in inflammation, ECM deposition, hypoxia, and angiogenesis. Also, they were considered as the key markers for studying the antirenal fibrosis effects of NE-THCQ. Thus, we speculated that the antirenal fibrosis effect of THCQ might be associated with its regulation of inflammation, hypoxia and ECM deposition by targeting TNF, IL-6, IL-1β and PI3K/AKT/mTOR and HIF-1α/VEGF signaling pathways with their relevant activators.

### Experimental Validation

#### Cytotoxicity Study of NE-THCQ in HK-2 Cells

**Figure 4:**
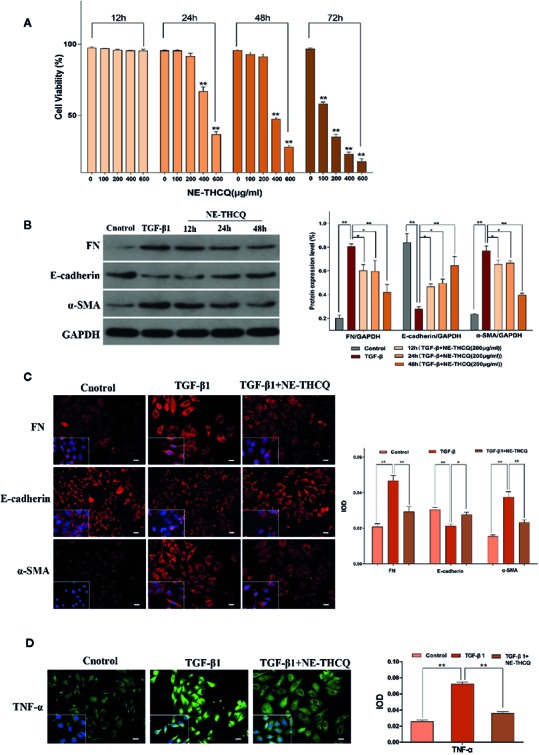
N-butanol extract of Taohe-Chengqi decoction (NE-THCQ) reversed epithelial-mesenchymal transformation (EMT) and inhibited extracellular matrix (ECM) accumulation and inflammation in TGF-β1-stimulated HK-2 cells. **(A)** NE-THCQ treatment cytotoxicity in HK-2 cells. The cytotoxicity of NE THCQ was determined by CCK-8 assay. **(B)** Effect of NE-THCQ on the expressions of FN, E-cadherin, and a-SMA. **(C)** The representative images and statistical graph of immunofluorescence staining for FN, E-cadherin, and α-SMA (400 ×). Bar = 20 μm. **(D)** The representative images and statistical graph of immunofluorescence staining for tumor necrosis factor-α (TNF-α) (400 ×). Bar = 20 μm. Data were presented as mean standard deviation (SD) of three independent experiments. *P < 0.05, **P < 0.01.

The viability of HK-2 cells was inhibited dose- and time-dependently after incubating with NE-THCQ at the concentrations of 0, 100, 200, 400, and 600 µg/ml for 12 h, 24 h, 48 h and 72 h (**Figure 4A**). The cell viability was significantly inhibited after NE-THCQ treatment at the concentration of 400 µg/ml for 24 hours (*P* < 0.01), and when the duration of incubation was increased (48 h, 72 h), the cell viability was significantly inhibited (*P* < 0.01). When 100 µg/ml NE-THCQ cultured for 72 hours, the cell viability was significantly inhibited (*P* < 0.01), and with the increase of concentration (200, 400, 600 µg/ml), the inhibitory effect of the cell viability was more obvious (*P* < 0.01). Thus, NE-THCQ was used at the concentration of 200 µg/ml for 12 h, 24 h, and 48 h for investigating its antirenal fibrosis effect.

#### NE-THCQ Inhibits ECM Synthesis and EMT Induced by TGF-β1 in HK2 Cells

To determine whether NE-THCQ affects the conversion of tubular epithelial cells into myofibroblasts and inhibits ECM synthesis *in vitro*, we used TGF-β1 (a well-characterized profibrogenic cytokine) to stimulate HK-2 cells, and α-SMA, E-cadherin, FN were also studied. Considering the cytotoxicity, 200 µg/ml concentration of NE-THCQ was selected as a suitable intervention concentration for subsequent experiments. Western blot analysis indicated that expression levels of α-SMA and FN were significantly increased, whereas those of E-cadherin was significantly decreased in the TGF-β1-treated cells (*P* < 0.01) (**Figure 4B**). NE-THCQ abrogated the TGF-β1-induced expression of α-SMA and FN, and simultaneously restored E-cadherin expression in HK-2 cells (*P* < 0.01 or *P* < 0.05) (**Figure 4B**). When the treatment time was prolonged, the effect of NE-THCQ on these genes was more significant at 48 h (*P* < 0.01). Thus, NE-THCQ (200 µg/ml) was treated for 48 h in further experiments. Immunofluorescent staining confirmed that the expression of α-SMA and FN were significantly increased, while E-cadherin was decreased in TGF-β1-induced cells, compared to the control group (*P* < 0.01). NE-THCQ treatment for 48 h in TGF-β1-induced HK-2 cells successfully reduced α-SMA and FN expression (*P* < 0.01) and upregulated the expression of E-cadherin (*P* < 0.05) (**Figure 4C**).

#### NE-THCQ Decreases the Expression of TNF-α in TGF-β1-Stimulated HK-2 Cells

Immunofluorescence study was used to measure the proinflammatory factor TNF-α. Compared to the control group, the expression of TNF-α was significantly increased in TGF-β1 induced HK-2 cells (*P* < 0.01), while the expression level of TNF-α in NE-THCQ treatment group was decreased (*P* < 0.01) (**Figure 4D**).

#### The Effect of NE-THCQ on TGF-β1-Induced F-Actin Assembling of HK-2 Cells

**Figure 5:**
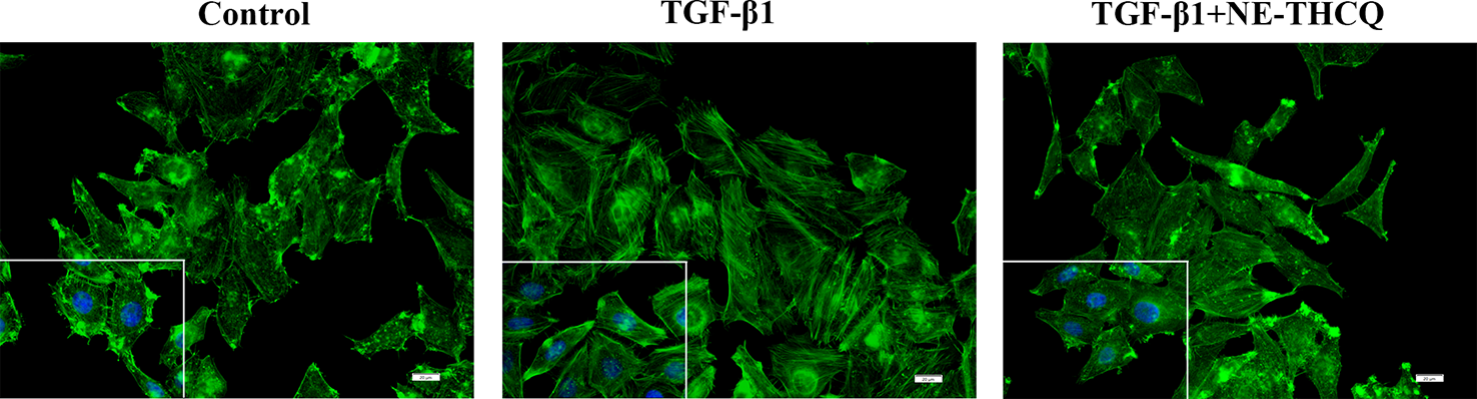
Representative images of F-actin assembling (400 ×). Phalloidin Alex-488 (green) staining showed the F-actin morphology and distribution in HK-2 cells. Bar = 20 μm.

During fibrosis, TGF-β1 can rearrange F-actin into parallel stress fibers, which promote EMT and myofibroblast activation. After phalloidin staining in **Figure 5**, TGF-β1 treatment induced a change in F-actin morphology and assembling in HK-2 cells. After treating NE-THCQ for 48 h, F-actin rearrangement was significantly reduced in TGF-β1 induced cells.

#### NE-THCQ Repressed PI3K/AKT/mTOR Signalling Along With HIF-1α/VEGF Signalling *In Vitro*

**Figure 6:**
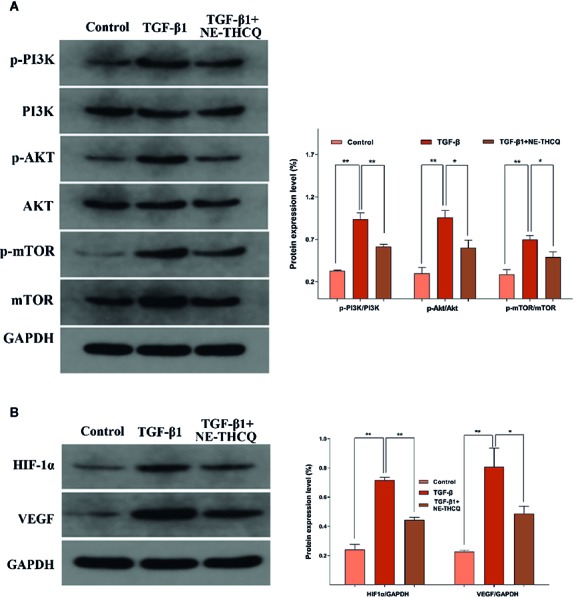
Experimental validation of the key signaling pathways *in vitro*. N-butanol extract of Taohe-Chengqi decoction (NE-THCQ) repressed the PI3K/AKT/ mTOR **(A)** and HIF-1a/VEGF **(B)** signaling pathways in HK-2 cells induced by TGF-β1. Data were presented as mean standard deviation (SD) of three independent experiments. *P < 0.05, **P < 0.01.

Network pharmacological analysis predicted that the molecular mechanism of the antirenal fibrosis effect of NE-THCQ might be highly associated with the pathways, including PI3K/AKT, mTOR and HIF-1α, VEGF signaling. We further validated the hypothesis of those potential targets and signaling pathways. According to **Figure 6A**, PI3K/AKT/mTOR signaling pathway was activated in TGF-β1 stimulated HK-2 cells. Compared to the control group, the protein levels of phosphorylated PI3K/AKT/mTOR were significantly increased (*P* < 0.01), but they were significantly repressed after NE-THCQ treatment for 48 h (*P* < 0.05 or *P* < 0.01). Likewise, HIF-1α and VEGF expression levels significantly increased after TGF-β1 stimulation (*P* < 0.01), and NE-THCQ treatment reduced the levels of HIF-1α (*P* < 0.01) and VEGF (*P* < 0.05) (**Figure 6B**)

#### The Effect of NE-THCQ on Renal Histopathology After UUO

**Figure 7:**
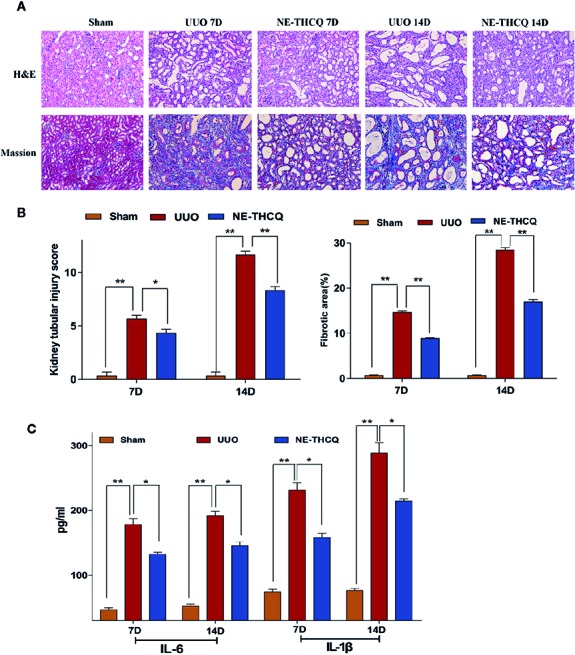
**(A)** Representative renal histopathological sections on the 7th and 14th day after unilateral ureteral obstruction (UUO) induction (200 ×). Renal tubular injury was assessed by hematoxylin-eosin (H&E) staining. Formation of fibrosis in kidney tissue was ascertained from Masson’s trichrome stained. **(B)** Kidney tubular injury scores based on H&E staining and relative fibrotic area (%) based on Masson’s trichrome staining. **(C)** Serum interleukin-1β (IL-1β) and IL-6 concentrations were tested by ELISA on 7th and 14th day after UUO. Data were presented as mean standard deviation (SD), n = 6 rats per group on the 7th or 14th day after UUO. *P < 0.05, **P < 0.01.

We determined the impact of NE-THCQ on renal injury and fibrosis after UUO induction *in vivo*. H&E staining revealed that the kidneys of rats represented diffuse inflammatory cell infiltration and interstitial edema on the 7th day after UUO induction. Fibrotic changes began on the 7th day after exposure to UUO, and severe renal interstitial fibrosis was noticed on the 14th day after UUO induction (*P* < 0.01) (**Figures 7A, B**). NE-THCQ could protect the UUO-injured kidney tissue from the inflammatory damage and progressive fibrosis on the 7th and 14th day, compared to the UUO group (*P* < 0.05 or *P* < 0.01). Similarly, Masson’s trichrome staining indicated that with prolonged obstruction period, UUO group exhibited a gradually increased content of collagen fibers (*P* < 0.01), while the extracellular matrix (ECM) deposition was significantly reduced by the NE-THCQ treatment on the 7th and 14th day (*P* < 0.01) (**Figures 7A, B**).

#### NE-THCQ Reduces the Releases of IL-6 and IL-1β in UUO Rats

Inflammation triggers renal fibrosis. Using the preliminary data analyses, we examined the proinflammatory cytokines selected from **Figure 3C**, including IL-6 and IL-1β. As predicted, with the prolongation of obstruction time, IL-6 and IL-1β levels were increased gradually in comparison with the Sham group (*P* < 0.01) (**Figure 7C**), while the increased levels were decreased after NE-THCQ treatment (*P* < 0.05).

#### NE-THCQ Attenuates ECM Deposition and EMT Induced by UUO

**Figure 8:**
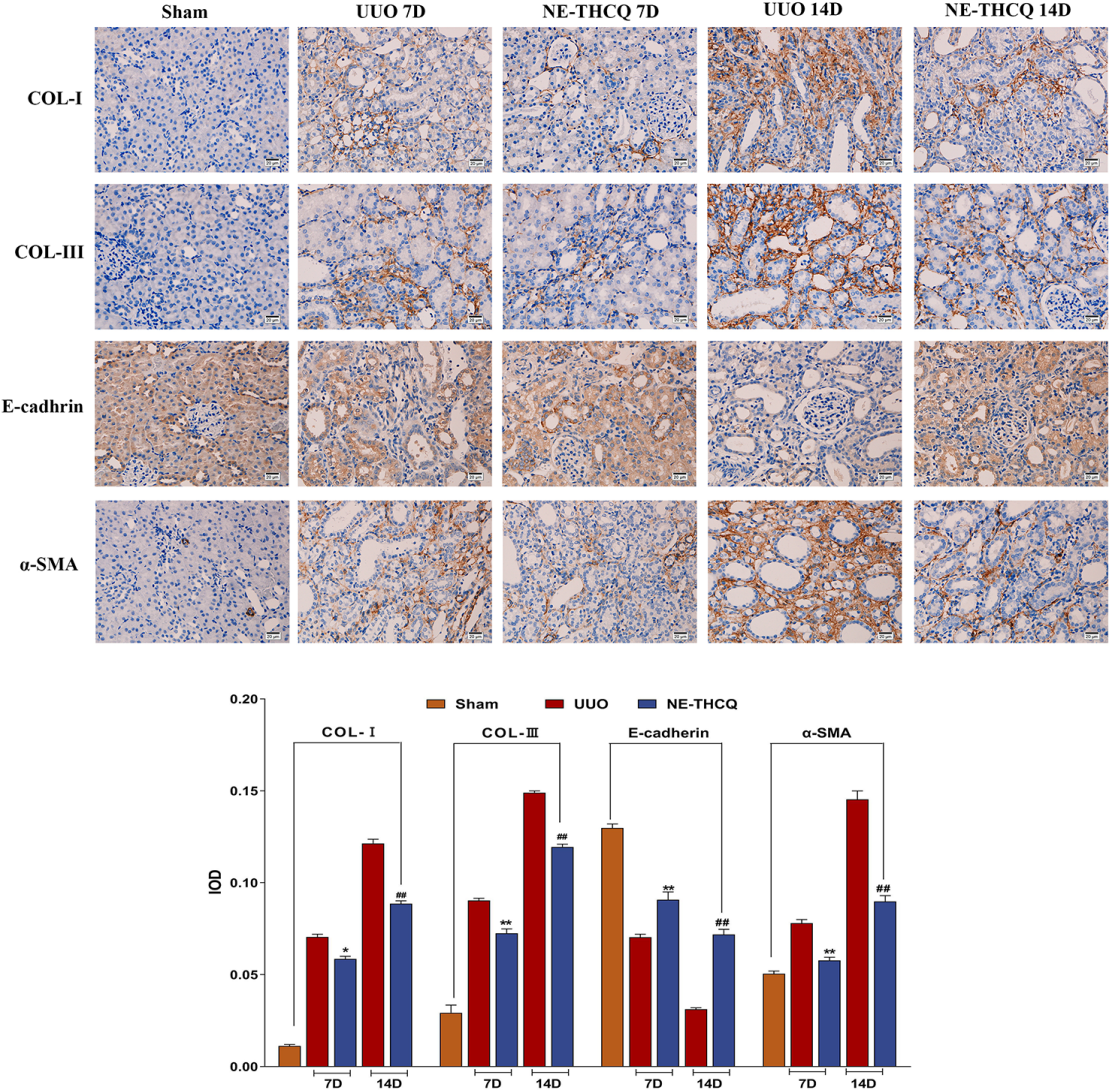
N-butanol extract of Taohe-Chengqi decoction (NE-THCQ) inhibited extracellular matrix (ECM) accumulation and epithelial-mesenchymal transformation (EMT) in the kidneys of unilateral ureteral obstruction (UUO) rats. The representative images and statistical graph of immunofluorescence staining for COL-I, COL-III, E-cadherin and α-SMA (400 ×). Bar = 20 μm. Data were presented as mean standard deviation (SD), n = 6 rats per group. For the NE-THCQ group vs. the UUO group on the 7th day, * indicates *P* < 0.05, ** indicates *P* < 0.01. For the NE THCQ group vs. the UUO group on the 14th day, ^##^ indicates *P* < 0.01.

After confirming the positive effect of NE-THCQ on EMT and ECM synthesis *in vitro*, we performed a further *in vivo* validation. In **Figure 8**, the expression levels of Col-I, Col-III, and α-SMA, as determined by immunohistochemical analysis, demonstrated gradual increases with the prolonged obstruction time, but E-cadherin expression decreased in the UUO group (*P* < 0.01). The semi-quantitative analysis showed that compared to the UUO group, Col-I, Col-III, and α-SMA levels were significantly reduced by the NE-THCQ treatment on the 7th and 14th day after modeling, while E-cadherin increased (*P* < 0.01 or *P* < 0.05).

#### NE-THCQ Improved the UUO-Induced Mitochondrial Morphological Injury

**Figure 9:**
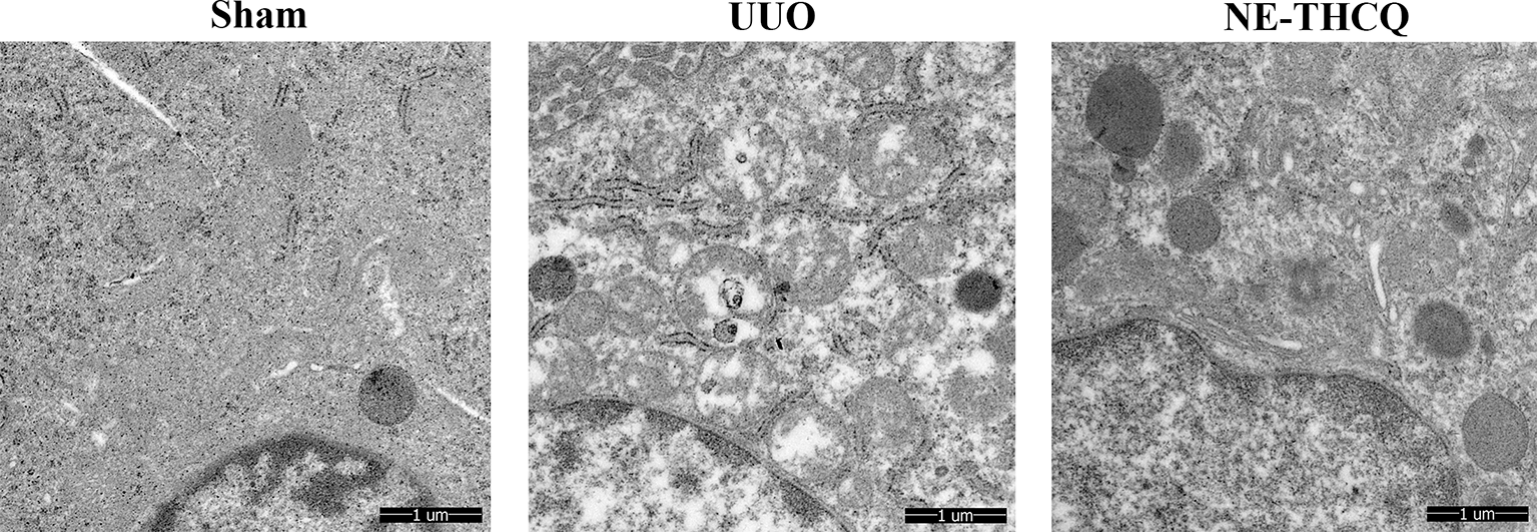
N-butanol extract of Taohe-Chengqi decoction (NE-THCQ) protected the mitochondrial structure in unilateral ureteral obstruction (UUO)–induced kidneys. Representative images of transmission electron microscopy (5000 ×). Bar = 1 μm.

NE-THCQ improved the mitochondrial morphological injury *in vivo*, as observed under a TEM, where the mitochondria were vacuolated and swollen in UUO treated rats compared to the Sham group (**Figure 9**). After treating NE-THCQ for 14 days, fewer mitochondrial swelling or accumulation of disrupted cristae architectures was noticed.

#### NE-THCQ Inhibits PI3K/AKT/mTOR and HIF-1α/VEGF Signalling Pathways in UUO Rats

**Figure 10:**
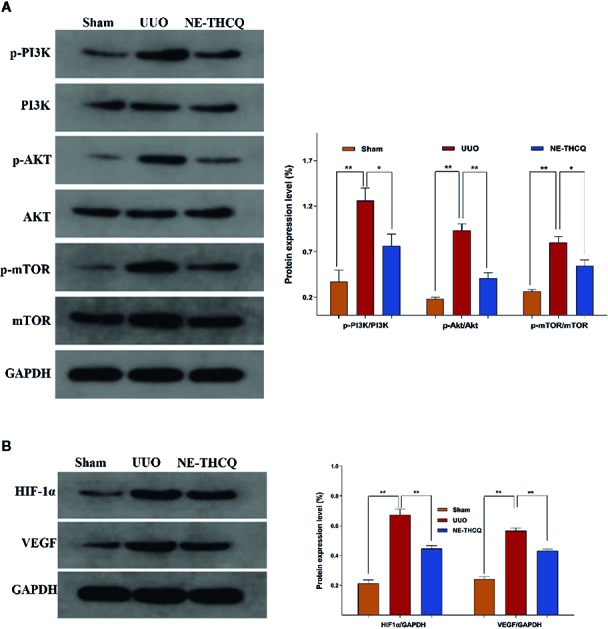
Experimental validation of the key signaling pathways *in vivo*. N-butanol extract of Taohe-Chengqi decoction (NE-THCQ) inhibited PI3K/ AKT/mTOR **(A)** and HIF-1α/VEGF **(B)** signaling pathways in the kidneys of unilateral ureteral obstruction (UUO) rats. Data were presented as mean standard deviation (SD) of three independent experiments. *P < 0.05, **P < 0.01.

As the target signaling pathways were verified *in vitro*, we carried out further *in vivo* validation. In **Figure 10**, Western blot analysis indicated that the level of phosphorylated PI3K/AKT/mTOR and the expression of HIF-1α and VEGF were upregulated in the UUO group than the Sham group (*P* < 0.01). After NE-THCQ treatment for 14 days, PI3K/AKT/mTOR signaling pathways were significantly suppressed compared to the UUO group (*P* < 0.01 or *P* < 0.05). Likewise, the expression levels of HIF-1α and VEGF was significantly reduced by the NE-THCQ treatment (*P* < 0.01).

## Discussion

Progressive renal fibrosis directly leads to ESRD. Because of the complex mechanisms, multiple protein targets, and pathways involved during the development and progression of renal fibrosis, a single targeted drug cannot achieve the desired therapeutic effect. The multicomponent-multitarget approach of TCM formula offers diverse therapeutic activities, but an in-depth study of the potential mechanisms of TCM formula is quite challenging. Although the chemical compositions of TCM are complex, not all compounds are pharmacologically active. TCM also contains toxic compounds, nonpharmacological compounds, and some impurities, while the contents of pharmacologically active compounds are generally low. Actually, TCM exerts its therapeutic effects through the synergistic effect of many pharmacologically active compounds (Li, 2017). Therefore, we resorted to the solvent extraction method to efficiently extract and separate the THCQ and finally obtained different polar fractions of THCQ. Sodium sulfate is prone to thermal decomposition; it is not decocted in the clinic but taken directly after dissolving. Sodium sulfate is soluble in water but insoluble in ethanol. Hence, we treated Sodium sulfate directly as an extracted part. Compared to other extraction methods, the solvent extraction method is simpler and more precise.

Our previous studies have found that n-butanol extract was the effective fraction in THCQ on antirenal fibrosis. In this study, we aimed at elucidating the material basis of antirenal fibrosis and the mechanism of the n-butanol extract of THCQ. Additionally, we integrated UPLC-Q/TOF-MS/MS analysis, network pharmacology analysis, and experimental verification to systematically elaborate the pharmacological mechanisms of NE-THCQ against renal fibrosis.

We first analyzed the compounds of NE-THCQ using UPLC-Q/TOF-MS/MS, which showed 26 ingredients from *Glycyrrhiza glabra* L., *Rheum palmatum* L., and *Prunus persica* (L.) Batsch; some of these components exhibited therapeutic effect against chronic kidney diseases. Especially, some active components of *Rheum palmatum* L. and *Prunus persica* (L.) Batsch exhibited antiinflammatory properties in patients with renal fibrosis (Li et al., 2011; Xiang et al., 2015). Glycyrrhetic acid from *Glycyrrhiza glabra* L. exerts a nephroprotective effect through reducing the serum creatinine level and decreasing the interstitial fibrosis in animal models (Wang et al., 2007). Instead of herb databases, these 26 ingredients, identified by UPLC-Q/TOF-MS/MS analysis, were selected as the potential candidates for network pharmacology analysis.

We screened the active ingredients using ADME simulation parameters, especially the OB and DL parameters as the important evaluation indexes. Among the 26 ingredients, those with OB values ≥ 30% and DL values ≥ 0.18 were considered as pharmacologically active. In the compound-target network, the compounds with a higher degree were considered to be more significant. In this study, 7, 2′, 4′-trihydroxy-5-methoxy-3-arylcoumarin represented the highest degree accounting for the major therapeutic component in NE-THCQ, followed by licorice glycoside E, hederagenin, beta-sitosterol, liquiritin, gallic acid-3-O-(6′-O-galloyl)-glucoside, and 18α-hydroxyglycyrrhetic acid.

Integrating the network topological parameters with all the network analyses, we exhibited that NE-THCQ acted at multiple targets and multiple signaling pathways. We determined the hub targets of the active compounds, namely, AKT1, VEGFA, TNF, IL6, HIF-1α, IL-1β, mTOR, and FN1. These genes are associated with inflammation, ECM, hypoxia, and angiogenesis. Many studies confirmed that inflammatory response and ECM deposition could be the crucial mechanistic pathways for developing renal fibrosis and hypoxia injury (Fredrik and Lina, 2011). Thus, NE-THCQ may exert its antirenal fibrosis effect by inhibiting ECM deposition, reducing inflammation and regulating hypoxia *via* PI3K/AKT/mTOR and HIF-1α/VEGF signaling pathways. To further validate this postulation, we investigated the therapeutic effects of NE-THCQ on renal tubular epithelial cells *in vitro* and unilateral ureteral obstruction (UUO) model in rats *in vivo*. UUO animal model is a classic model to investigate renal fibrosis (Yu et al., 2020). In the present study, we ligated the left ureter of rats to establish UUO rats and chose 80 mg/kg NE-THCQ for animal study based on the preexperiment (**Supplementary Figure 2**).

The pathological characteristics of renal fibrosis include inflammatory infiltration, the disappearance of renal intrinsic cells and excessive deposition of the ECM. During the persistent renal injury, renal tubular epithelial cells display myofibroblast-like phenotype through multiple biological changes; this process is called epithelial-mesenchymal transformation (EMT). Once EMT is activated, the cells start to lose their epithelial markers (such as E-cadherin) and acquire the mesenchymal markers (such as α-SMA) and then promote ECM secretions (such as collagen and fibronectin) (Nieto et al., 2016; Attila et al., 2019). The *in vitro* Western blotting assay showed that NE-THCQ dramatically inhibited the expressions of FN and α-SMA and upregulated the E-cadherin level in TGF-β1 induced cells time-dependently. Then, we incubated with NE-THCQ for 48 h in further experiments. Immunofluorescence staining confirmed the same results. Cytoskeletal reorganization and increased mobility are the characteristics of EMT (Yu et al., 2019). We also identified that NE-THCQ could restrain actin fibers assembling and regulate the actin cytoskeleton. *In vivo*, we found that NE-THCQ attenuated renal fibrosis after UUO surgery, and this conclusion was supported by less pathological alterations in Masson’ s stain, lower expression of α-SMA, Col-I and Col-III and higher expression of E-cadherin in the NE-THCQ group compared to the UUO group. Inflammation is an early response to kidney injury. However, persistent inflammation promotes the synthesis of fibrogenic factors, thus leading to renal fibrosis (Xiao et al., 2014). Inflammatory cytokines, such as tumor necrosis factor-α (TNF-α), interleukin-1β (IL-1β), and IL-6, can directly mediate renal fibrosis through inflammatory infiltration, fibroblast activation and synthesis and degradation of ECM (Andrade et al., 2019; Gorjipour et al., 2019; Shrikant, 2019). Our results showed that NE-THCQ inhibited inflammatory infiltration in H&E staining analysis and reduced the levels of serum IL-1β and IL-6 *in vivo*. *In vitro* studies, immunofluorescence staining demonstrated that the TGF-β1 induced excessive production of TNF-α was repressed by NE-THCQ in HK-2 cells. Thus, as predicted by network pharmacology analyses, we validated that NE-THCQ could inhibit ECM synthesis, reverse EMT and repress inflammatory response *in vitro* and *in vivo*.

PI3K/AKT/mTOR signaling pathway is a crucial pathway in renal fibrosis. Phosphoinositide 3-kinase (PI3K) (a lipid kinase) can specifically catalyze the phosphorylation of phosphatidyl inositol-3 hydroxyl and produce inositol lipid with the second messenger effect (Manoj and John, 2019). PI3K primarily phosphorylates PIP2 to yield PIP3 and then activates AKT. Activated AKT can cause a series of phosphorylation cascade reactions and regulate vital downstream effector molecules, like mTOR to exert its biological effect (Dirk et al., 2018; Li et al., 2019). PI3K/AKT/mTOR signaling pathway controls cell growth, proliferation, cell polarity and cytoskeleton, and EMT and angiogenesis (Peng et al., 2014; Arash and Amirhossein, 2017; Falguni et al., 2018). Recent studies have shown that activated PI3K/AKT/mTOR signaling pathway can induce EMT process, especially E-cadherin level and inhibit matrix metalloproteinases (MMPs) (Karimi et al., 2019). MMPs can degrade almost all ECM components and E-cadherin, except polysaccharide (Oskar et al., 2019). Thus, the expression of MMPs can be upregulated by suppressing the PI3K/AKT/mTOR signaling pathway, which would inhibit the pathological deposition of ECM and EMT extension. Also, many animal experiments have proved that inhibition of mTOR signaling can markedly ameliorate renal interstitial inflammation, renal dysfunction and renal fibrosis associated with CKD (Wilfred and Jerrold, 2009). Additionally, mTOR can change the PKC phosphorylation state, then directly phosphorylate and activate AKT to regulate the cell’s actin skeleton and promote the EMT process (Wilfred and Jerrold, 2009; Maggie et al., 2018). Interestingly, our *in vitro* and *in vivo* studies demonstrated that NE-THCQ could inhibit the expression of the PI3K/AKT/mTOR signaling pathway.

Hypoxia injury is a common pathway of CKD and an initial factor of renal fibrosis (Liu et al., 2017). Fibrosis causes microvasculature injury and inflammatory stimulation decreases oxygen permeation and increases renal tubular epithelial cells metabolism, which can lead to the persistent hypoxic condition of renal tissue. Conversely, hypoxia can trigger the apoptosis of renal tubular epithelial cells and stimulate the phenotype transformation from renal tubular cells to myofibroblasts (Hu et al., 2017; Liu et al., 2018; Shu et al., 2019). Hypoxia could also promote the expression of tissue inhibitors of metalloproteinases (TIMPS), which would inhibit the degradation of ECM, reduce the blood flow of capillaries around renal tubules and damage the diffusion of oxygen, thus aggravating the local hypoxia (Volker, 2009). Among the various response pathways activated by hypoxia, the most important steps are mediated by hypoxia-inducible factors (HIFs). HIFs also upregulate the expression of proangiogenesis genes, especially VEGF. However, a dual role of VEGF has been proved in renal injury (Mayer, 2011). Conversely, the upregulation of VEGF promotes neovascularization to protect local renal tissue in the early stage of kidney hypoxia injury. The inflammatory response of continuous hypoxia injury in the renal interstitium of CKD might be associated with an increase in VEGF accompanied by the overgeneration of new blood vessels. HIF-1α, an active subunit of HIF-1, is mainly expressed in the cytoplasm and regulated by oxygen concentration. Therefore, we investigated the role of the HIF-1α/VEGF signaling in the pathogenesis of NE-THCQ against renal fibrosis. Our studies demonstrated that NE-THCQ also inhibited the HIF-1α/VEGF signaling pathway *in vitro* and *in vivo*. The TEM images demonstrated swelling and vacuolation in the morphology of mitochondria after UUO injury; NE-THCQ could attenuate the morphological changes in mitochondria by inhibiting HIF-1α/VEGF pathway to adapt to hypoxia injury. Interestingly, some studies suggest that HIF-1α is regulated by the mTOR signaling pathway, and mTOR increases the cellular levels of HIF-1α, which in turn stimulates the expression of angiogenic factors, such as VEGF, PDGF-A, and TNF-α (Wilfred and Jerrold, 2009). Finally, our findings suggest that NE-THCQ may inhibit renal fibrosis progression mainly by regulating the inflammatory process, reversing EMT and decreasing ECM deposition through PI3K/AKT/mTOR and HIF-1α/VEGF signaling pathways. However, further experiments should be conducted to investigate whether NE-THCQ could inhibit HIF-1α/VEGF pathway in PI3K/AKT/mTOR pathway-dependent manners.

## Conclusion

In this study, the active components and molecular mechanisms of NE-THCQ against renal fibrosis were illustrated by adopting an integrated strategy, which employed UPLC-Q/TOF-MS/MS analysis, network pharmacological analysis, and experimental validation. A total of 26 components of NE-THCQ was identified by UPLC-MS/MS study and adopted for further network pharmacology analysis. Seven active components were identified by simulating the ADME process. Through experimental validating the hub targets and hub signaling pathways by network pharmacological analysis, we demonstrated that NE-THCQ could suppress inflammatory and EMT processes and decrease abnormal ECM deposition through PI3K/AKT/mTOR and HIF-1α/VEGF signaling pathways for exerting its antirenal fibrosis effect. Additionally, our study also suggested that the combination of network pharmacology prediction and experimental validation effectively elucidated the multi-target and multilink pharmacological mechanisms of TCM formula.

## Data Availability Statement

The data used to support the findings of this study will be available from the corresponding author upon request.

## Ethics Statement

The animal study was reviewed and approved by the Ethics Committee of Hubei University of Traditional Chinese Medicine (Hubei, China). All experiments of animals were conducted in conformity with the Animal Care and Use Committee of Institute of Materia Medica, P.R. China.

## Author Contributions 

SZ and ZA designed and conducted the study with equal contribution. SZ drafted the original manuscript. YB revised the manuscript. PY, CW, and YB supervised the study. WL, LL, and YH contributed to the formal analysis and data curation. All authors approved the final manuscript.

## Funding

This study was supported by Academic Experience Inheritance of the Sixth National Group of Old Chinese Medicine Experts of the State Administration of Traditional Chinese Medicine (No. 2017 [29]) and the key projects of Hubei Provincial Department of Health (No. JX6A09).

## Conflict of Interest

The authors declare that the research was conducted in the absence of any commercial or financial relationships that could be construed as a potential conflict of interest.

## Supplementary Material

Figure S1 Total ion chromatogram of n-butanol extract of THCQ.

Figure S2 Pictures and datas of preexperiment.
