# Supplementary figures and images for: Deciphering the Pharmacological Mechanisms of Taohe-Chengqi Decoction Extract Against Renal Fibrosis Through Integrating Network Pharmacology and Experimental Validation In Vitro and In Vivo
Source: Front Pharmacol. 2020 Apr 16;11:425. doi: 10.3389/fphar.2020.00425 (PMC7176980; doi:10.3389/fphar.2020.00425)

## Positive

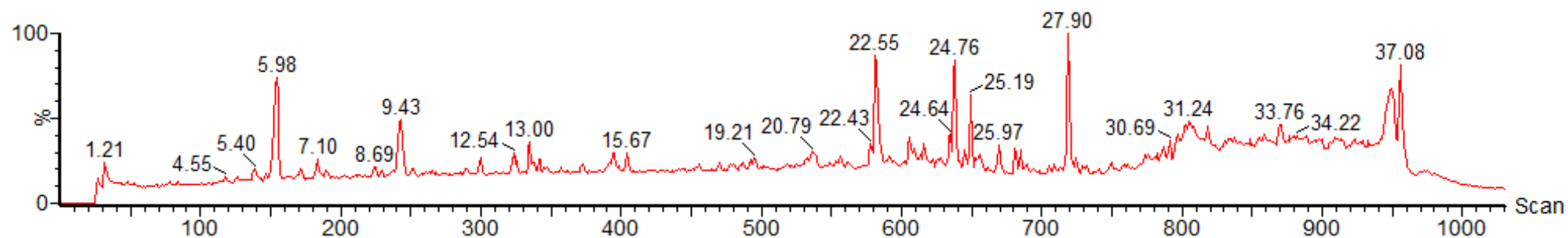

## Negative

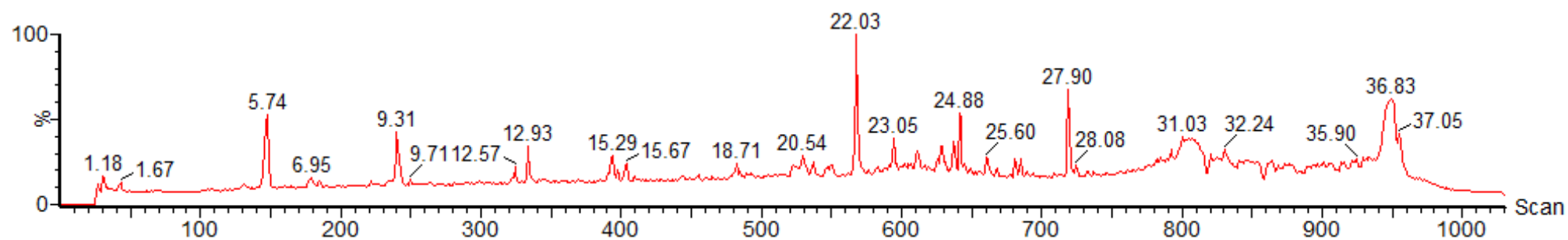

Supplementary Figure 1

Supplement: Figure S1 — Total ion chromatogram of n-butanol extract of THCQ. [file Image_1.pdf]

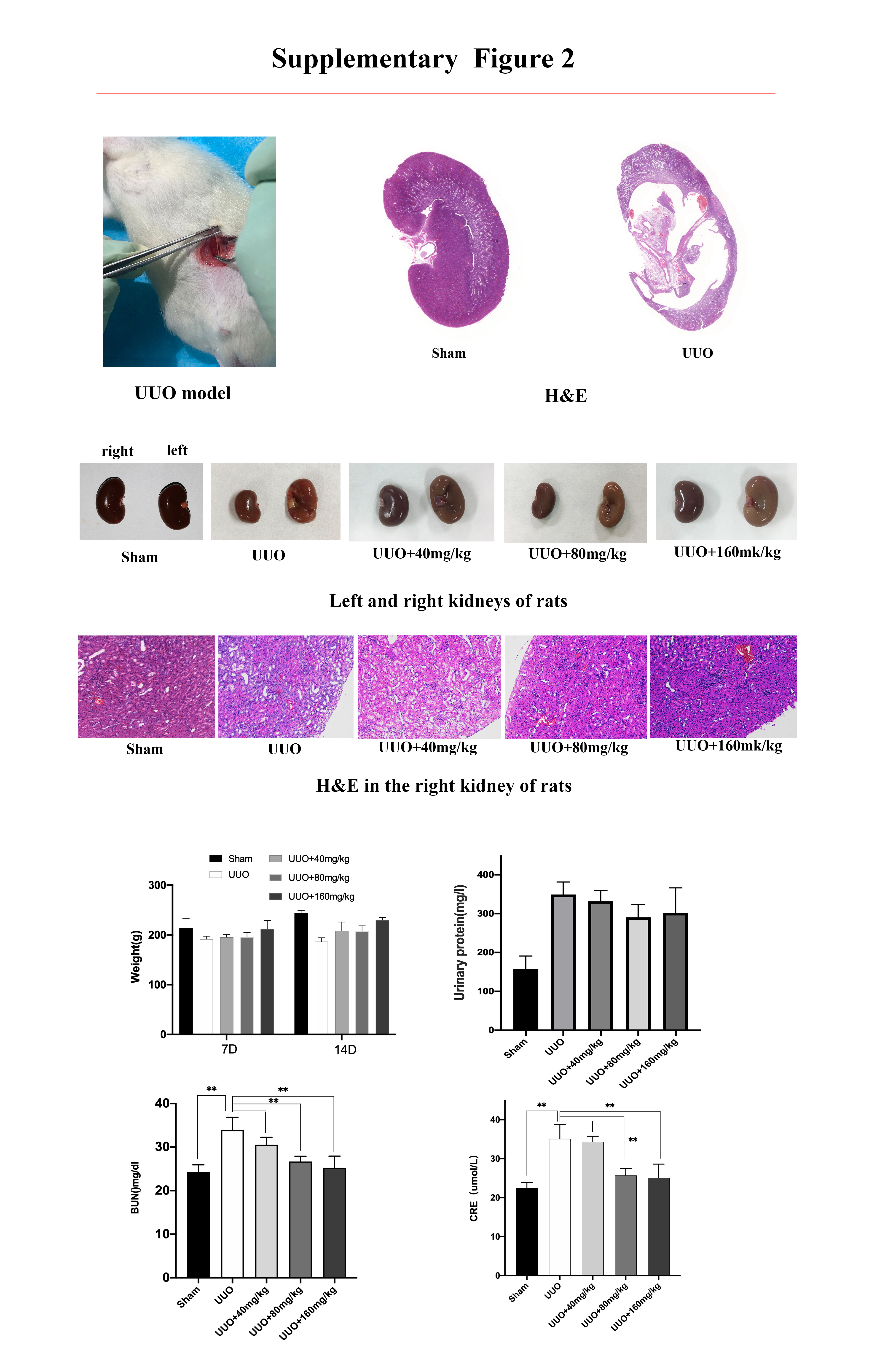

Supplement: Figure S2 — Pictures and datas of preexperiment. [file Image_2.jpg]
